# Tetrodotoxins (TTXs) and *Vibrio alginolyticus* in Mussels from Central Adriatic Sea (Italy): Are They Closely Related?

**DOI:** 10.3390/md19060304

**Published:** 2021-05-25

**Authors:** Simone Bacchiocchi, Debora Campacci, Melania Siracusa, Alessandra Dubbini, Francesca Leoni, Tamara Tavoloni, Stefano Accoroni, Stefania Gorbi, Maria Elisa Giuliani, Arianna Stramenga, Arianna Piersanti

**Affiliations:** 1 Istituto Zooprofilattico Sperimentale Umbria e Marche “Togo Rosati”, Via Cupa di Posatora, 3, 60131 Ancona, Italy; d.campacci@izsum.it (D.C.); m.siracusa@izsum.it (M.S.); a.dubbini@izsum.it (A.D.); f.leoni@izsum.it (F.L.); t.tavoloni@izsum.it (T.T.); s.accoroni@staff.univpm.it (S.A.); a.stramenga@izsum.it (A.S.); a.piersanti@izsum.it (A.P.); 2 Dipartimento di Scienze della Vita e dell’Ambiente, Università Politecnica delle Marche, Via Brecce Bianche, 60131 Ancona, Italy; s.gorbi@staff.univpm.it (S.G.); m.e.giuliani@staff.univpm.it (M.E.G.)

**Keywords:** tetrodotoxins (TTXs), mussels, *Vibrio alginolyticus*, HILIC-MS/MS, PKS gene, NRPS gene, Adriatic Sea, bacterial pellets

## Abstract

Tetrodotoxins (TTXs), potent neurotoxins, have become an increasing concern in Europe in recent decades, especially because of their presence in mollusks. The European Food Safety Authority published a Scientific Opinion setting a recommended threshold for TTX in mollusks of 44 µg equivalent kg^−1^ and calling all member states to contribute to an effort to gather data in order to produce a more exhaustive risk assessment. The objective of this work was to assess TTX levels in wild and farmed mussels (*Mytilus galloprovincialis*) harvested in 2018–2019 along the coastal area of the Marche region in the Central Adriatic Sea (Italy). The presence of *Vibrio* spp. carrying the non-ribosomal peptide synthetase (NRPS) and polyketide synthase (PKS) genes, which are suspected to be involved in TTX biosynthesis, was also investigated. Out of 158 mussel samples analyzed by hydrophilic interaction liquid chromatography coupled with tandem mass spectrometry (HILIC-MS/MS), 11 (7%) contained the toxins at detectable levels (8–26 µg kg^−1^) and 3 (2%) contained levels above the EFSA safety threshold (61–76 µg kg^−1^). Contaminated mussels were all harvested from natural beds in spring or summer. Of the 2019 samples, 70% of them contained *V. alginolyticus* strains with the NRPS and/or PKS genes. None of the strains containing NRPS and/or PKS genes showed detectable levels of TTXs. TTXs in mussels are not yet a threat in the Marche region nor in Europe, but further investigations are surely needed.

## 1. Introduction

Tetrodotoxins (TTXs) are potent neurotoxins, which have been known for centuries and have been implicated worldwide in pufferfish poisoning. Structurally, TTXs are alkaloids with a guanidinium moiety connected to a highly oxygenated carbon skeleton [1,2,3,4]. The guanidinium group is responsible for their toxicity; it binds to the voltage-gated Na^+^ channel pores on neuronal and muscle cell membranes and thus blocks nervous signal transmission [5,6]. After eating contaminated seafood, symptoms of tetrodotoxin poisoning progress from tongue and lip numbness to progressive paralysis and, in the worst cases, death as a result of respiratory failure.

Tetrodotoxin (TTX) is the best known member of the group, but it co-exists with some other natural occurring congeners. There have been 30 structural analogues reported to date, with different degrees of toxicity depending on their chemical structure [7,8].

TTX is over a thousand time more toxic to humans than cyanide, with a lethal dose of 2–3 mg (corresponding to 40–60 µg kg^−1^ b.w.) [9,10]. Besides pufferfish, other fish of the Tetraodontidae, Didontidae, and Molidae families, along with organisms phylogenetically quite distant such as bivalve mollusks, gastropods, sea slugs, echinoderms, crabs, and ribbon worms, are known to harbor TTXs [11,12,13,14,15,16,17].

In the attempt to explain the genesis of TTXs, the endogenous theory, namely the ability of TTX-containing organisms to produce TTXs independently, has gradually lost its appeal over the years [4,18,19,20] leaving room for the exogenous theory, which explains contamination of marine species with TTXs as a result of symbiotic bacterial synthesis [21,22]. The bacterial genus most commonly reported to be associated with TTX production is *Vibrio,* followed by *Bacillus*, *Pseudomonas*, *Actinomyces*, and *Micrococcus* [23,24,25,26]. Among the *Vibrio* species capable of producing TTXs, *V. alginolyticus* and *V. parahaemolyticus* are the most frequently reported [21,27,28].

The TTX biosynthetic pathway has still not been elucidated, but several hypotheses have been proposed. Kotaki et al. [29] theorized the inclusion of guanidinium moiety in the TTX molecule to be the result of a synthetic pathway in which arginine is transformed by an amidinotransferase (AMT) and nonribosomal peptidesynthetase (NRPS) module. Chau et al. [4] hypothesized TTX to be assembled by a hybrid polyketide synthase (PKS)/NRPS enzyme complex, possibly incorporating an AMT. Despite none of these hypotheses having been experimentally proven, it is likely that the NRPS and PKS gene products play an important role in TTX biosynthesis. NRPS and PKS genes, therefore, have been targeted in the protocols for detection of potential TTX-producing bacteria [28].

Until recently, TTXs were considered to be confined to the Indo-Pacific area, especially Japan, where pufferfish are caught and eaten as fugu, a traditional culinary delicacy. Fugu consumption in Japan results in several tens of severe intoxications every year, with fatal outcomes in some cases [30]. The Japanese regulations for food safety specify a limit of 2 mg TTX equivalent kg^−1^ [10].

Since 2003, pufferfish, which probably entered through the Suez Canal, has been reported in the Eastern Mediterranean Sea [31,32]. In 2006, the first European case of TTX intoxication occurred in Spain in a patient who ate gastropods (*Charonia lampas lampas*) from Portugal [33]. Following that episode, several cases of TTX shellfish contamination have been reported in the UK, Greece, the Netherlands, and Spain since 2015 [27,34,35,36].

On the basis of these events, TTXs are now considered the emerging toxins most threatening food security in Europe; nevertheless, maximum permitted limits have not yet been set, nor specific monitoring plans adopted. The only reference to TTXs in European legislation can be found in the ban on the sale of poisonous Tetraodontidae and derived products [37]. In 2017, the European Food Safety Authority (EFSA) published the scientific opinion piece “Risks for public health related to the presence of tetrodotoxin (TTX) and TTX analogues in marine bivalves and gastropods” [10], wherein a preliminary guidance level of 44 µg TTX equivalent kg^−1^ was proposed as a reference for member states. In the same document, more data were requested in order to provide a better exposure assessment. Following this publication, TTXs were detected in Italian shellfish from Syracuse Bay (Sicily) and Marano Lagoon (Friuli Venezia Giulia) [38,39].

This work aimed to study the presence of TTXs in mussels (*Mytilus galloprovincialis*) from the Marche region, Central Adriatic Sea (Italy), and the possible correlation with symbiotic vibrios over a two-year period (2018–2019). Mussels were analyzed using hydrophilic interaction liquid chromatography coupled with tandem mass spectrometry (HILIC-MS/MS) to measure TTX contamination. A sampling campaign was undertaken to detect any geographical or seasonal trends. Moreover, the most contaminated samples were dissected and the digestive glands analyzed separately to study the distribution of TTX in mollusk tissues. Bacterial strains belonging to the *Vibrio* genus were isolated from the samples and tested via polymerase chain reaction (PCR) to confirm the *Vibrio* species and to detect the presence of two potential pathway genes, PKS and NRPS. The strains carrying one or both genes were cultured and analyzed by HILIC-MS/MS to assess for possible TTX synthesis.

The results of this work fulfill the call for data from the EFSA to EU member states, provide useful information for a better understanding of TTX origin, and describe a study of TTX accumulation in marine organisms.

## 2. Results

### 2.1. Method Performance Assessment

The developed HILIC-MS/MS method showed good analytical performance. Matrix-matched calibration curves exhibited good linearity with correlation coefficients greater than 0.99 and response factor drift < 10%.

The limit of detection (LOD) was 8 µg kg^−1^ and the limit of quantification (LOQ) was 26 µg kg^−1^ for all the matrices included in the present study. The sensitivity was not excellent, due to the significant matrix effect (signal suppression of 70–80%) and the instrument features; the system can be classified as a low-sensitivity system, as described by Turner et al. [40]. However, the method was suitable to identify TTX contamination at the EFSA guidance level of 44 µg equivalent kg^−1^ for TTX.

Good recoveries (R%) of 99% and 97% and acceptable repeatability relative standard deviations (RSDr%) of 7% and 8% were obtained in mussels spiked at 75 µg kg^−1^ and 251 µg kg^−1^, respectively.

Internal quality controls carried out within the different analytical batches were in agreement with the validation parameters.

### 2.2. TTXs in Mussels

#### 2.2.1. Year 2018

Of the 99 samples of mussels (*Mytilus galloprovincialis*) analyzed during 2018 using HILIC-MS/MS, 96 (97%) did not show the presence of TTX above the method LOD (8 µg kg^−1^), and only 3 (3%) were contaminated with levels between the LOD and LOQ (>8 and <26 µg kg^−1^) (Table S1).

A rough calculation of the contamination levels by single-point comparison with a standard at LOQ level resulted in a value of ~10 µg kg^−1^. All the contaminated samples contained only TTX itself. None of the eight other analogues included in the method were detected (<LOD). The contaminated samples were harvested during July from natural Pesaro beds (VA180705W, VA180731W, and MS180731W). None of the farmed samples showed detectable TTX (>LOD) (Table S1).

#### 2.2.2. Year 2019

**Figure 1:**
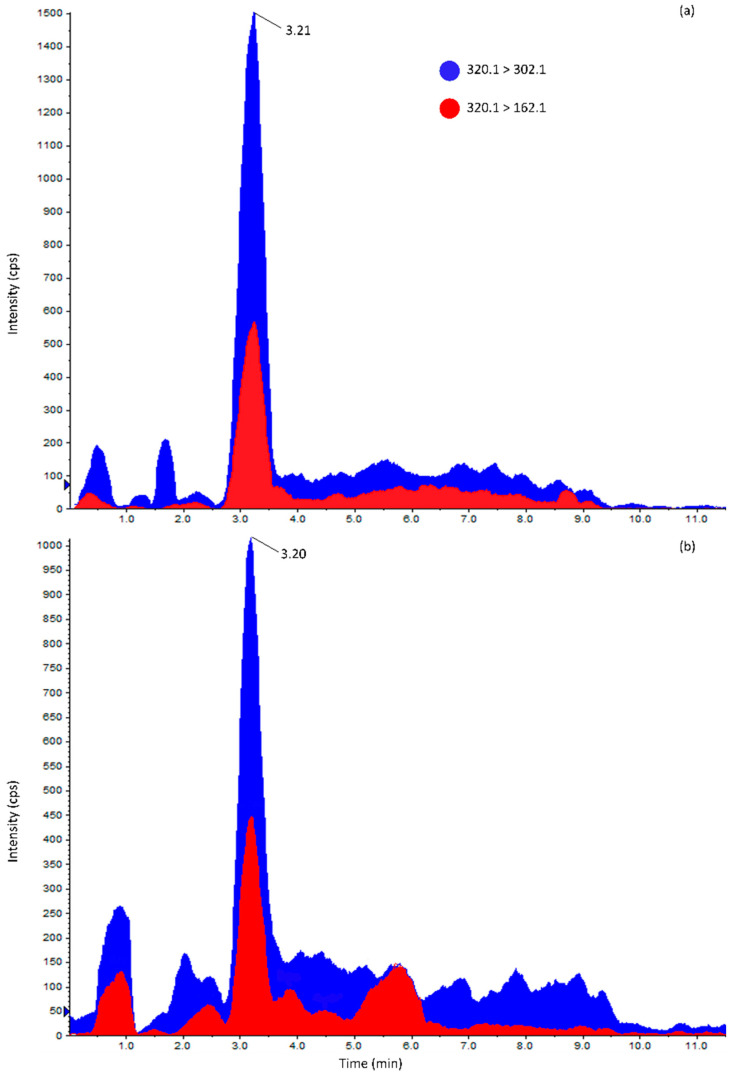
HILIC-MS/MS chromatograms of the (**a**) TTX matrix-matched standard (65 ng mL^−1^); (**b**) digestive gland of the field sample SN190702W (196 µg kg^−1^). 320.1 > 302.1, multiple reaction monitoring (MRM) transition for TTX quantification. 320.1 > 162.1, MRM transition for TTX confirmation.

Out of the 59 samples analyzed, harvested between May and early September from Pesaro and Ancona natural beds, 48 (81%) were below the LOD while 11 (19%) showed detectable TTX levels (>8 µg kg^−1^). Among the latter group, 8 showed contamination levels between the LOD and the LOQ (>8 and <26 µg kg^−1^) and 3 showed levels above the LOQ (>26 µg kg^−1^; Table S1). The majority of TTX-containing samples (9 of 11) and the most contaminated ones came from the Ancona area (Table S1 and Figure 1). Sample AS190605W contained 67 µg kg^−1^ of TTXs, while SN190702W and SS190702W contained 76 and 61 µg kg^−1^, respectively.

As regards the Pesaro area, only two samples were contaminated, and both showed a concentration just above the LOD (>8 µg kg^−1^).

All the contaminated samples were harvested from mid-June to the beginning of August and, as in 2018, only the parent toxin TTX was detected. Six of the seven sampling points gave at least one sample contaminated at a detectable level.

#### 2.2.3. Distribution of TTX in Mussel Tissues

All the samples containing detectable amounts of TTX were dissected and the digestive glands were analyzed using HILIC-MS/MS. Assuming a mussel composition of 20% by weight for the digestive gland (DG) and 80% for the remaining flesh (RF), a homogenous distribution of toxin between tissues would result in 20% of toxin in the DG and 80% in RF.

The six samples with TTX levels between the LOD and LOQ showed similar contamination levels in the DG and the whole flesh (WF), indicating a uniform distribution of the toxin. In sample AS190605W, characterized by significant TTX levels, a 3-fold higher concentration of toxin was reported in the WF (67 µg kg^−1^) compared to the DG (~22 µg kg^−1^) indicating that roughly 93% of the total TTX was in the RF and only 7% in the DG (Table S2).

Among the samples with the highest TTX levels, SN190702W and SS190702W showed 2.6-fold and 1.7-fold higher concentrations of TTX in the DG (196 and 107 µg kg^−1^, respectively) than the WF (76 and 61 µg kg^−1^, respectively) suggesting a preferential accumulation of the toxin in the DG (52% and 35% of the total contamination, respectively).

#### 2.2.4. Uptake and Depuration Rate Estimation

**Figure 2:**
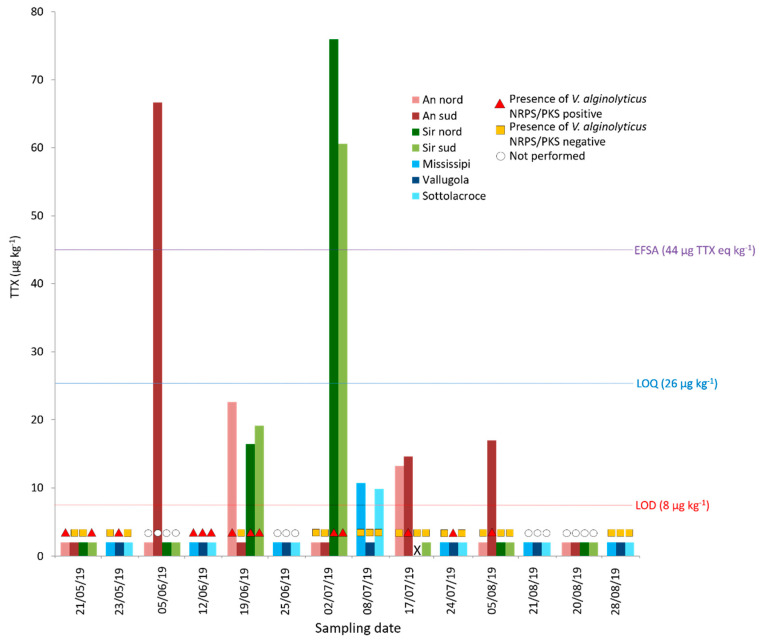
TTX concentrations and *Vibrio alginolyticus* in mussels collected from Pesaro and Ancona natural beds during 2019. X = not analyzed for TTXs.

In the Ancona sampling area, the toxin concentrations recorded and, in some sites, the sampling frequency, allowed the estimation of TTX uptake and depletion rates. In the An sud site, two weeks before the positive TTX sample identification (67 µg kg^−1^), no traces of the toxin were detected (Figure 2).

At the Sir nord and Sir sud sites, no TTX was detectable one month before the maximum levels were reached (76 µg kg^−1^ and 61 µg kg^−1^, respectively). Two weeks before, values of ~18 and ~23 µg kg^−1^ (just below the LOQ) were detected at the two sites respectively. At all the sampling points, the TTX reached at least 85% clearance two weeks after having reached the highest contamination (Figure 2). At the An nord and An sud, TTX showed more than one maximum in the contamination profile during the 2019 sampling campaign, which lasted four months.

### 2.3. Vibrio Characterization

#### 2.3.1. Year 2018

**Figure 3:**
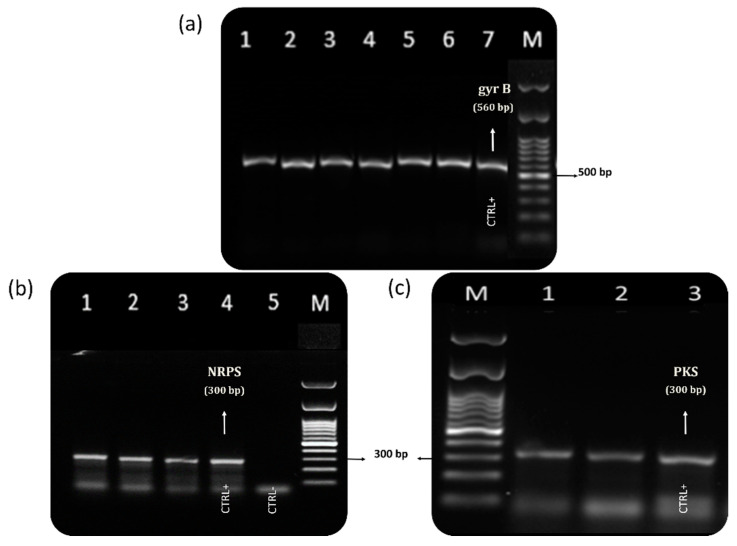
Agarose gel electrophoresis of PCR products of (**a**) gyr B (560 bp), (**b**) NRPS (300 bp), and (**c**) PKS (300 bp) genes in *Vibrio alginolyticus* strains isolated from mussels collected from the coast of the Marche region. Lane 1–6 in (**a**), 1–3 in (**b**), and 1–2 in (**c**) represent field samples; lane 7 in (**a**) represents the positive control of *V. alginolyticus* ATCC 33787(CTRL^+^). Lanes 4 in (**b**) and 3 in (**c**) represent the positive controls of *V. parahaemolyticus* ATCC 17802 (CTRL^+^); lane 5 in (**b**) represents the negative control (CTRL^−^). M = molecular weight marker.

Eighty-one of the 99 samples of mussels collected during 2018 were also analyzed using a culturing method to assess the presence of *Vibrio* spp. *V*. *alginolyticus* was isolated and identified in 50 (62%) of the 81 samples analyzed using PCR targeting a species-specific marker (Table S1 and Figure 3), while *V. parahaemolyticus* was never detected. All *V. alginolyticus* isolates were analyzed by PCR to detect the presence of NRPS and PKS genes. *V. alginolyticus* colonies harboring NRPS or PKS genes were isolated from three (4%) samples: the NRPS gene was detected in two isolates (3% of the samples), while PKS was detected in only 1 (1%). These three mussel samples were harvested from farming plants during spring or summer, and TTXs were not detected in any of them. All isolates bearing the NRPS or PKS genes were cultured and analyzed for TTXs using HILIC-MS/MS. None of them showed detectable levels of the toxins.

#### 2.3.2. Year 2019

In 2019, *Vibrio* spp. isolation and identification were carried out on 35 mussel samples (Table S1), and in all of them *V. alginolyticus* strains were isolated and identified by PCR targeting a species-specific marker. NRPS and/or PKS genes were detected by PCR in *V. alginolyticus* isolates from 14 (40%) of the samples. Isolates from eight (23%) samples harbored only the NRPS gene and one (3%) sample only the PKS, while isolates from five samples (14%) carried both genes. All isolates harboring NRPS and/or PKS genes were from samples (14) harvested between May and August, but TTXs were detected in only seven of them.

All the sampling points returned at least one isolate of *V. alginolyticus* containing NRPS and/or PKS gene.

#### 2.3.3. TTX and NRPS/PKS-Positive *Vibrio alginolyticus* in Mussels Sampled in 2019

Aiming to study the possible coexistence of NRPS/PKS-positive *Vibrio alginolyticus* and TTX contamination, further observations focused on the mussels collected during 2019 from natural beds, during the warmer seasons (spring and summer). The latter factors seemed to promote both TTX mussel contamination and NRPS/PKS-positive *Vibrio alginolyticus* isolation.

In 2019, 11 mussel samples contained TTX > LOD. Ten of them were tested for *Vibrio* spp. (one contaminated sample was not tested) and seven (70%) were found to be positive for *V. alginolyticus* carrying the NRPS and/or PKS genes. Conversely, 14 mussel samples containing *V. alginolyticus* with the NRPS and/or PKS genes were analyzed for TTX and seven (50%) showed detectable levels. NPRS and/or PKS genes were always present in the samples with high TTX levels (≥15 µg kg^−1^) (Table S1, Figure 2 and Figure 3).

## 3. Discussion

Across the whole study, out of 158 mussel samples analyzed for TTXs, in 144 (91%) the toxin levels were lower than the LOD, in 11 (7%) they were between the LOD and LOQ, and only 3 (2%) of them showed levels between 61 and 76 µg kg^−1^.

Looking at Europe, the EFSA has reported that of the more than 1600 results provided from Great Britain, Greece, and the Netherlands on TTXs in bivalve mollusks collected between 2006 and 2016, 92% showed no contamination or levels below 25 µg kg^−1^, and in only 32 samples TTXs were above the recommended level (between 47 and 253 µg kg^−1^) [10].

In a recent study conducted in the Galician Rias (Spain) during 2017, TTXs were found in only 2 of 1279 samples of mollusks, at levels around 0.9 µg kg^−1^ [28]. In France, of 127 bivalve mollusk samples collected during 2018, only 4 showed detectable TTX levels (1.7–11.2 µg kg^−1^) [41].

In Italy, TTXs were measured at 0.8–6.4 µg kg^−1^ in 14 out of 25 shellfish collected in spring and summer from 2015 to 2017 in Syracuse Bay (Sicily) [38]. Exceptionally high contamination levels were reported in the Marano Lagoon (North Adriatic Sea), where TTX levels were measured in mussels of 541 µg kg^−1^ in 2017 and 216 µg kg^−1^ in 2018 [39].

Therefore, the results reported herein for mussels from the Marche coast are comparable with the European ones. The literature data show that TTXs in bivalve mollusks are not yet a serious threat to consumer health, even if contamination levels above the EFSA recommendation of 44 µg kg^−1^ were detected in a small percentage of samples.

A more thorough analysis of the 2018 Marche coastal data showed undetectable TTX levels (<LOD) in all the farmed samples and only slight contamination in three mussels from Pesaro natural beds. In long-line mussel farms, mollusks live in pelagic waters (1–2 nautical miles from the coast) with high hydrodynamics and depths often above 5 m. Wild mussels live clinging to rocks, near the coast, and generally in shallow water. The contaminated mussels in the present study were harvested in July, a month characterized by high solar radiation and water temperatures around 25 °C. These environmental conditions are in agreement with the results of Turner et al. [42], who reported a clear relationship between the incidence of TTX-contaminated bivalve mollusks in Great Britain and the environmental characteristics of sampling sites. They pointed out that most of the contaminated samples came from sites characterized by shallow water (<5 m), relatively low salinity, and high temperature (>15 °C). Leao et al. [28] reported that among bivalve mollusk samples taken from Galicia between January and September 2017, those contaminated by low levels of TTXs came from intertidal areas, with water temperatures close to or above 15 °C and medium–low salinity.

All the above results confirmed the hypothesis that Marche mussels from natural beds may be more prone to TTX contamination than farmed ones. Since high temperature also seems to be a causative factor, the sampling campaign in 2019 was focused in spring and summer, and only on natural beds, to increase the likelihood of finding TTX-contaminated mollusks. It turned out that in 2019, a significantly higher percentage (14% vs. 3% in 2018) of samples showed detectable TTX levels.

Despite the small number of contaminated samples and the low levels measured, an attempt to evaluate TTX uptake and depuration rates was made for specific sampling sites. In general, TTX accumulation in mussels seems to be not as fast as in the case of other marine biotoxins [43], while it seems that total toxin clearance always occurs in a timespan of two weeks or less (Figure 2). Moreover, at some sampling sites, more than one maximum and subsequent drop in contamination were recorded during the biweekly sampling intervals. All these findings confirm the previous reports by Turner et al. [42] for bivalves from the United Kingdom.

Even though TTX levels above/around the LOQ were measured in the DG and WF of only three mussel samples out of nine, some consideration of tissue distribution may be attempted. The compartmentalization study highlighted three possible patterns: (i) despite the low TTX levels, the less contaminated samples showed a substantially uniform distribution of toxin between DG and RF; of the three more highly contaminated samples, (ii) one (AS190605W) showed preferential TTX accumulation in RF (93% of total TTX content), while (iii) the other two (SN190702W and SS190702W) showed preferential accumulation in DG (52% and 35%, respectively).

Preferential accumulation in DG has been previously reported for other marine biotoxins in mollusks [44,45].

The TTX distribution pattern in the mollusk tissues has previously suggested a hypothesis of different possible routes of exposure [46]. Accumulation in the DG could indicate dietary TTX uptake, while a more homogeneous distribution in the mollusk tissues could be the result of contamination from symbiotic microorganisms. However, it has also been demonstrated that after ingestion and accumulation in the DG, TTXs can migrate from one tissue compartment to another in bivalve mollusks [47]. High toxin accumulation in DG can thus provide evidence for recent or ongoing TTX contamination.

Interestingly, the two samples with the highest contamination levels in DG (SN190702W and SS190702W) were harvested on the same day from two neighboring sampling sites with very similar environmental conditions. We can also hypothesize for them the same TTX exposure time.

Few data are available in the literature about TTX distribution in mollusk tissues. Vlamis et al. [34] found similar levels in the DG and WF (202.9 and 179.1 µg TTX kg^−1^, respectively) of Greek mussels, while Biessy et al. [46,47,48] reported preferential accumulation of TTXs in siphons and DG in clam species from New Zealand (*Paphies australis*). Rapkova et al. [49] found the highest TTX levels in the DG of Pacific oysters (*Crassostrea gigas*) harvested from a production area in southern England. It also seems that different bivalve species differ in tissue accumulation patterns; therefore, the reported compartmentalization in mussels from the central Adriatic Sea may contribute to better understanding of the TTX compartmentalization behavior.

*Vibrio* spp. isolation and identification in mussels from the Marche coast showed a high incidence of *V. alginolyticus*, with 50 contaminated samples out of 81 analyzed (62%) in 2018 and 35 out of 35 (100%) in 2019.

These results were partially expected because vibrios are among the most abundant bacteria in the marine environment [50] and *V. alginolyticus* is the predominant species along the Italian Adriatic coast, followed by *V. parahaemolyticus*, *V. cholerae*, and *V. vulnificus* [51]. The increased incidence observed in the 2019 samples compared to those from 2018 is the result of sampling site (natural beds) and sampling period (spring–summer) selection in the second year of the monitoring campaign. It is known that the occurrence of *Vibrio* spp. is positively correlated with temperature, especially in temperate regions [52]. Additionally, the 2019 improvement of the *Vibrio* isolation method could have increased the analysis sensitivity.

Moreover, the incidence of *V. alginolyticus* with NRPS and/or PKS genes was significantly higher in 2019 (40%) than in 2018 (4%). Again, the explanation for this evidence may be found in the positive correlation between the presence of these genes in vibrio strains and the warm sampling season. Furthermore, the three *V. alginolyticus* strains with NRPS and/or PKS genes found during 2018 were isolated from mussels harvested between May and July. The correlation between the presence in vibrios of genes responsible for other toxins, such as thermostable direct hemolysin (*tdh*) and thermostable direct hemolysin-related hemolysin (*trh*), and the warm season has been previously reported [53]. In the future, more evidence should be collected on the correlation between the presence of *V. alginolyticus* carrying NRPS and PKS and the environmental temperatures.

None of the NRPS- and/or PKS-positive strains showed detectable production of TTXs. It has previously been reported that bacterial TTX production, although proven, is very variable in terms of rate (from less than 1 ng mL^−1^ of extract to a few hundred) [23] and not always easily detectable. Furthermore, several studies have reported that the NRPS and PKS pathway genes in bacteria are not always related to TTX production, which probably occurs only in specific conditions or as a result of certain unknown stimuli [21]. It has also been hypothesized that some TTX-producing bacteria tend to lose their ability to synthesize the toxin if cultured in an artificial medium [23,54].

The focused mussel survey conducted during 2019 may enable some consideration of the possible coexistence of TTX contamination and *V. alginolyticus* carrying NRPS and/or PKS. A total 70% (7 out of 10) of the mussels that showed TTX > LOD (and analyzed for *V. alginolyticus*) and 100% of the samples with estimated concentrations > 15 µg/kg harbored *V. alginolyticus* carrying the NRPS and/or PKS genes. Conversely, in the 50% of mussels containing *V. alginolyticus* with the NRPS and/or PKS genes, TTX was measured at a detectable level (>LOD). These findings highlight the concomitance between TTX contamination and NRPS/PKS-positive *V. alginolyticus* in mussels from the Marche region. However, to confirm the possible correlation between the two parameters, longer-term studies are needed (Figure 2).

## 4. Materials and Methods

### 4.1. Sampling

**Figure 4:**
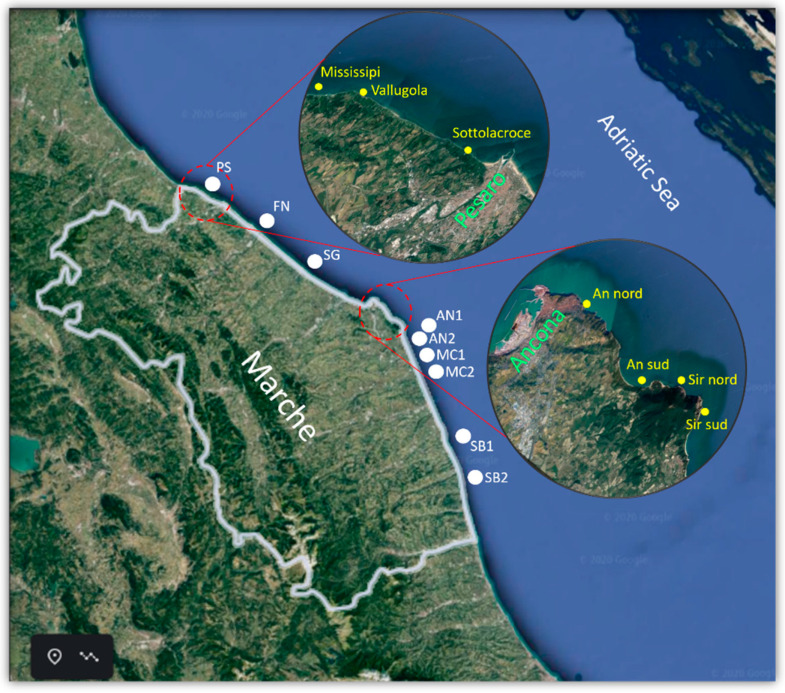
*Mytilus galloprovincialis* sampling sites along the Marche coast. Nine breeding sites: PS (PS), FN (Fano), SG (Senigallia), AN1 (Ancona 1), AN2 (Ancona 2), MC1 (Macerata 1), and MC2 (Macerata 2), SB1 (San Benedetto del Tronto 1), SB2 (San Benedetto del Tronto 2). Seven natural beds: Mississipi, Vallugola, Sottolacroce in the Pesaro area, An nord, An sud, Sir nord, and Sir sud in the Ancona area.

A total of 158 mussel samples (*Mytilus galloprovincialis*) were collected between 2018 and 2019 from Marche region harvesting areas included in the biotoxin regional monitoring plan (Central Adriatic Sea, Italy). Nine were breeding areas and seven were wild sites (Figure 4).

Natural mussel beds in the Marche region show the unique features of a high, jagged and rocky coast, an exception in the Adriatic Sea, surrounded by straight sandy coasts. In 2018, samples were collected once a month from January until the end of August; in summer, sampling frequency was increased to twice a month. In 2019, the sampling was limited to the seven wild sites from May until early September, generally at a biweekly frequency. Each sample consisted of about 40–50 commercially sized mussels (5–7 cm) with a weight of approximately 4 kg. Samples were submitted both to chemical and microbiological analyses.

### 4.2. Chemical Analysis

#### 4.2.1. Chemicals and Standards

Acetonitrile (LC-MS grade) and methanol (HPLC grade) were purchased from CARLO ERBA Reagents S.r.l. (Cornaredo, Milan, Italy). Ammonium hydroxide (≥25% in water, LC-MS grade) was purchased from Merck (Darmstadt, Germany), formic acid (LC-MS grade) from VWR International (Radnor, PA, USA), and glacial acetic acid (reagent grade) from Sigma-Aldrich (Steinheim, Germany). Ultrapure water was produced by the MilliQ water purification system (Millipore Ltd., Bedford, MA, USA).

Tetrodotoxin Certified Reference Material (CRM-003-TTXs) was obtained from Cifga Laboratory (Lugo, Spain). The CRM was a mixture of tetrodotoxin (25.9 ± 1.2 µg g^−1^) and 4,9-anhydro tetrodotoxin (2.99 ± 0.16 µg g^−1^).

A stock solution in water was prepared from the CRM. This solution was used to obtain calibration standards and to spike the blank samples used in quality control and in method validation.

#### 4.2.2. TTX Extraction from Mussels and Bacterial Pellets

The extractions were performed following the EU-SOP “Determination of Tetrodotoxin by HILIC-MS/MS” [55], without the clean-up step. Moreover, the extraction protocol for bacterial pellets was implemented as described by Turner et al. [27] with minor changes. The details are described below.

- Mussels (WF, DG)

Bivalves were opened immediately once they arrived in the laboratory. Sand and solid residues were removed under running water, and the mussels were taken out of the shells and drained on a net. For each sample, about 150 g of WF was pooled and finely homogenized. DGs were dissected, pooled (10–15 specimens), finely homogenized, and analyzed separately in order to investigate the distribution of TTXs in the mussel tissues.

WF or DG homogenate (5.0 ± 0.1 g) was extracted with 5 mL of acetic acid (1% *v*/*v*), vortex-mixed for 3 min, and placed in a boiling water bath (100 °C) for 5 min. The extract was cooled to room temperature, vortex-mixed for 3 min, and centrifuged at 3000× *g* for 10 min. Supernatant (1 mL) was transferred to a microcentrifuge tube, to which 5 μL ammonium hydroxide was added and the sample was vortex-mixed for 3 min and centrifuged at 10,000× *g* per 1 min. The final extract was diluted (1:2) with a solution of acetonitrile (80% *v*/*v*) containing acetic acid (0.25% *v*/*v*), filtered through a 0.2 μm syringe filter and analyzed by HILIC-MS/MS.

- Bacterial pellets

Bacterial cultures (400 mL) were centrifuged at 3000× *g* for 30 min and the pellets (about 1 g) were collected in 50 mL PP centrifuge tubes. Each pellet was extracted with 1 mL of acetic acid (1% *v*/*v*) in a boiling water bath (100 °C) for 5 min, and then cooled to room temperature and centrifuged at 3000× *g* for 15 min. The supernatant was diluted (1:2) with a solution of acetonitrile (80% *v*/*v*) containing acetic acid (0.25% *v*/*v*), filtered through a 0.2 μm syringe filter, and analyzed by HILIC/MS-MS.

#### 4.2.3. HILIC-MS/MS Analysis

The chromatographic separation was achieved according to the EU-SOP “Determination of Tetrodotoxin by HILIC-MS/MS” [55]; details are given in Table S3. HILIC chromatography requires additional chromatographic methods for equilibration, column cleaning, and shutdown (Table S3).

**Figure 5:**
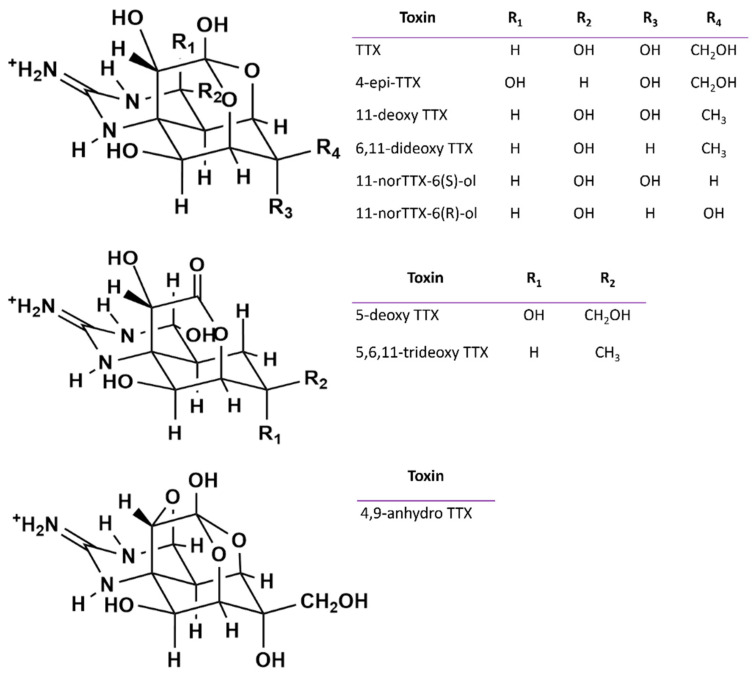
Molecular structure of TTX analogues monitored via the HILIC-MS/MS method.

Mass spectral experiments were performed using a hybrid triple-quadrupole/linear ion trap 3200 Q TRAP mass spectrometer (AB Sciex, Darmstadt, Germany) equipped with a Turbo V source and an electrospray ionization (ESI) probe. The mass spectrometer was coupled to a 1200-HPLC (Agilent—Palo Alto, CA, USA), which included an in-line degasser (G1379B), a quaternary pump (G1311A), a refrigerated autosampler (G1329A), and a column oven (G1316A). Infusion and flow injection experiments were performed on TTX CRM to optimize compound-dependent and ion source parameters. Nine analogues (Figure 5) were monitored via multiple reaction monitoring (MRM), with two transitions selected for each toxin to allow correct quantification and identification.

The MS acquisition method is described in Table S3. The unequivocal identification of the TTX chromatographic peak was accomplished by retention time comparison and ion ratio verification for the two characteristic mass transitions in the samples and in a matrix-matched standard. All the other analytes, for which no reference materials were available, were identified by selecting specific transitions from literature. The calibration curves were matrix-matched in order to deal with the relevant matrix effect. All analogues were then quantified with TTX, assuming an equimolar response.

#### 4.2.4. Analytical Method Performance Assessment

Method performances were investigated through in-house validation experiments in mussels, focusing on TTX, which was the only CRM available.

Instrumental linearity was investigated via matrix-matched calibration curves on six concentrations, (6.5, 13, 19, 26, 65, 130 ng mL^−1^), prepared in triplicate and run for intra-laboratory reproducibility. The mussels used for matrix-matched calibration curve preparation showed TTX levels < LOD. The calibration curves (y = bx + a) were obtained by plotting the toxin’s chromatographic peak areas (y) against concentrations (x). The best-fit curves were obtained by using the least-squares regression model. Linearity was evaluated from the correlation coefficients and response factor variation.

LOD and LOQ were estimated as the concentrations giving S/N ratios of 3 and 10, respectively, for the least intense (qualifier) transition monitored. The LOQ was calculated from the lowest calibration level and the LOD used was derived from the LOQ by dividing by 3.3. Subsequently, the estimated LOQ was experimentally confirmed by spiking blank mussel samples with the TTX CRM. The calculated LOD was extended to all the other TTX analogues.

Throughout the study, concentrations >LOD and <LOQ were reported, but we were aware of dealing with numbers affected by a larger uncertainty than if they were >LOQ since their variability was above the Horvitz–Thomson theoretical equation prescriptions. These numbers may be an estimation of possible contamination levels, enabling consideration of uptake and detoxification.

Accuracy in terms of R% and precision in terms of intraday repeatability (RSDr%) were assessed by replicated analyses (N = 6) on blank mussel samples spiked at 75 µg kg^−1^ and 251 µg kg^−1^ (TTX levels often found in European shellfish). The spiked samples were quantified against the matrix-matched calibration curves.

#### 4.2.5. Quality Control

Internal quality controls were included in sample analysis: a blank mussel sample was spiked with TTX at the LOQ in each analytical batch. Accuracy in terms of R% was calculated to check validation performances.

All the samples in which TTX was quantified were subjected to co-chromatography, in which they were spiked with a comparable amount of TTX to indubitably confirm the analyte identification.

### 4.3. Microbiological Analysis

#### 4.3.1. Isolation of *Vibrio* spp. from Mussel Samples

Bivalve mollusk samples were transported to the laboratory in refrigerated conditions and processed immediately upon receipt. For microbiological analysis of *Vibrio*, samples were externally cleaned with potable water and prepared for analysis in accordance with ISO 6887-3 [56]. In aseptic working conditions, about 10 individuals were opened and the flesh meat and intervalvular fluids were pooled together. Briefly, 25 g of bivalve sample was weighed, 225 mL of alkaline saline peptone water (ASPW) was added, and the sample was homogenized in a blender and incubated at 37 °C for 24 ± 3 h. After incubation, enrichment broths were subcultured onto selective media, Thiosulfate citrate bile sucrose agar (TCBS) and CHROM™ agar *Vibrio* (CHROMagar, France). These subcultures were further incubated at 37 °C for 24 ± 3 h. After incubation, colonies were selected on the basis of distinctive morphology and color. At least five yellow and/or green colonies from each TCBS plate, and mauve, blue, and white colonies from each CHROM™ agar *Vibrio* plate were isolated; colonies were subcultured on Tryptic soy agar (TSA) with 3% NaCl and identified via molecular assay.

In 2019, the samples of *Vibrio* spp. were isolated according to ISO 21872-1:2017 [57,58].

#### 4.3.2. Mass Culture of Bacterial Isolates

Bacterial isolates that were positive for NRPS and PKS genes according to PCR were cultured in 3% NaCl sterile nutrient broth with a final volume of 400 mL. Cultures were incubated at 25 °C with constant shaking (250 rpm) for 3 days, centrifuged at 3220× *g* for 15 min, and the pellets were finally collected for chemical analysis as described above [21,59].

#### 4.3.3. DNA Extraction—Operative Method

Bacterial colonies were suspended in 500 µL sterile distilled water, heated to 99 °C for 10 min, and centrifuged at 13,200 rpm for 1 min [60]. The supernatant was either tested by PCR immediately or stored at −20 °C. Possible *V. alginolyticus* colonies were submitted to PCR analysis for the species-specific *gyrB* gene [61] while those of *V. parahaemolyticus* were analyzed for the species-specific *toxR* gene [62].

#### 4.3.4. PCR Analysis

All the PCR amplifications were accomplished with a Mastercycler pro Thermal Cycler (Eppendorf).

- *gyrB and toxR* species-specific genes

Suspected *V. alginolyticus and V. parahaemolyticus* colonies were submitted to PCR analysis for detection of the species-specific *gyrB* [61] and *toxR* [62] genes, respectively. PCR amplification protocols are described in Table S4. The primers Alg F1 and AlgR1 (Invitrogen-Themo Fisher) were employed for the amplification of the *gyrB* gene fragment (568 bp) [61], while ToxR-F and ToxR-R primers were used for the detection of the *toxR* gene fragment (368 bp) [62]. *V. alginolyticus* ATCC 33787 and *V. parahaemolyticus* ATCC 17802 strains (American Type Culture Collection, Manassas, VA, USA) were used as positive controls of amplification (CTRL^+^) for *gyrB* and *toxR*, respectively, in all analytical batches. Ultrapure distilled nuclease-free water was used as a negative amplification control (CTRL^−^). The bacterial isolates were identified as *V. alginolyticus* or *V. parahaemolyticus* when PCR amplification generated products of the expected size by comparison to a 100 bp DNA ladder molecular weight marker and the positive control strain, after visualization by electrophoresis in 1.5% agarose gel under UV light.

- NRPS and PKS biosynthesis genes

The bacterial isolates identified as *V. alginolyticus* or *V. parahaemolyticus* were subjected to PCR analysis for the presence of PKS and NRPS genes. PCR amplification protocols are described in Table S4. The primers A2gamF and A3gamR were employed for the amplification of the NRPS gene fragment (300bp) [63]. DKF and DKR degenerate primers were used for the amplification of PKS gene fragment (300 bp) [28,64]. *V. parahaemolyticus* ATCC 17802 strain was used as a positive control of amplification (CTRL^+^) for both target genes in all analytical batches. Ultrapure distilled nuclease-free water was used as a negative amplification control (CTRL^−^). The *V. alginolyticus* and *V. parahaemolyticus* isolates were considered to be NRPS and/or PKS positive when PCR amplification generated products of the expected size by comparison to the molecular weight marker and the positive control strain, after visualization by electrophoresis in 1.5% agarose gel under UV light.

## 5. Conclusions

TTX in mussels from the Marche region coasts, Central Adriatic Sea, seems to not yet be a threat, with very few samples showing levels above the EFSA recommended threshold. The conducted survey showed that natural beds during the warmer seasons are the sites most prone to contamination. This evidence is in agreement with previous findings across various European countries, including Italy. *V. alginolyticus* containing NRPS and/or PKS genes seems to play a role in TTX accumulation in mussels, but further investigations are still needed. The present paper adds information which may help to better understand causative factors, uptake/detoxification rates, and TTX tissue distribution in mussels.

## Supplementary Materials

The following are available online at https://www.mdpi.com/article/10.3390/md19060304/s1, Table S1: TTX and *Vibrio* spp. contamination in mussel samples from coastal area of the Marche region in 2018–2019, Table S2: TTX distribution in digestive gland (DG) and remaining flesh (RF) of contaminated mussels., Table S3: HILIC-MS/MS method for TTX analysis: chromatographic conditions, MS parameters, and MRM transitions, Table S4: Reagents and protocols for *Vibrio* spp. PCR analysis.
